# Feasibility of an Intergenerational Program Supporting Healthy Aging for People Living With Traumatic Brain Injury

**DOI:** 10.1093/geroni/igaf122.3772

**Published:** 2025-12-31

**Authors:** Pascale Simard, Megan Veilleux, Valérie Poulin, Marie-Christine Ouellet, Marie-Eve Lamontagne, Krista Best, Thierry Belleguic, Samuel Turcotte

**Affiliations:** Université Laval, Québec, Quebec, Canada; Université Laval, Québec, Quebec, Canada; Université du Québec à Trois-Rivières, Trois-Rivières, Quebec, Canada; Université Laval, Québec, Quebec, Canada; Université Laval, Québec, Quebec, Canada; Université Laval, Québec, Quebec, Canada; Université Laval, Québec, Quebec, Canada; Université Laval, Québec, Quebec, Canada

## Abstract

**Introduction:**

Individuals living with traumatic brain injury (TBI) experience barriers to social participation, limiting healthy aging. While intergenerational programs may offer a solution, their feasibility with the TBI population remains unexplored. Using a community-engaged approach, we co-developed and delivered an intergenerational TBI program in a community organization dedicated to TBI health promotion. Intergenerational dyads (n = 8) partnered with professional photographers over seven structured sessions, sharing their thoughts or experiences about aging with disability, while turning words into art. The program culminated in a traveling art exhibition, launched at a national museum.

**Objectives:**

(1) To explore perceived program impacts, and (2) to assess feasibility and acceptability.

**Method:**

Post-intervention, we conducted semi-structured interviews with 15 TBI participants, a community worker, and the director, using a qualitative descriptive design guided by the Theoretical Framework of Acceptability. Data were analyzed with mixed methods and reported per the COREQ checklist.

**Results:**

TBI dyad participants experienced greater social participation, felt more valued and empowered, and perceived increased public awareness of their realities. The program reached TBI members who had not been involved with the organization before, creating opportunities for meaningful social connections. Participants valued and supported the program, but improving its flexibility to diverse participants’ abilities would enhance its feasibility.

**Conclusion:**

Co-creating and delivering inclusive health promotion initiatives like an art-based intergenerational program for individuals living with TBI is feasible and can promote social connection, participation and healthy aging.

